# Fabrication and Evaluation of Quercetin Nanoemulsion: A Delivery System with Improved Bioavailability and Therapeutic Efficacy in Diabetes Mellitus

**DOI:** 10.3390/ph15010070

**Published:** 2022-01-05

**Authors:** Manohar Mahadev, Hittanahalli S. Nandini, Ramith Ramu, Devegowda V. Gowda, Zainab M. Almarhoon, Mohammed Al-Ghorbani, Yahia N. Mabkhot

**Affiliations:** 1 Department of Pharmaceutics, JSS College of Pharmacy, JSS AHER, Mysuru 570015, India; mmanohar@jssuni.edu.in; 2 Department of Pharmaceutics, NGSM Institute of Pharmaceutical Sciences, Nitte University, Mangaluru 575018, India; 3 Department of Pharmacology, JSS College of Pharmacy, JSS AHER, Mysuru 570015, India; nandinisk19@gmail.com; 4 Department of Biotechnology and Bioinformatics, Faculty of Life Sciences, JSS AHER, Mysuru 570015, India; ramith.gowda@gmail.com; 5 Department of Chemistry, College of Science, King Saud University, P.O. Box 2455, Riyadh 11451, Saudi Arabia; zalmarhoon@ksu.edu.sa; 6 Department of Chemistry, College of Science and Arts Ulla, Taibah University, P.O. Box 84901, Riyadh 11681, Saudi Arabia; mghorbani@taibahu.edu.sa; 7 Department of Chemistry, College of Education, Thamar University, Dhamar 87246, Yemen; 8 Department of Pharmaceutical Chemistry, College of Pharmacy, King Khalid University, P.O. Box 960, Abha 61421, Saudi Arabia; ygaber@kku.edu.sa

**Keywords:** quercetin, nanoemulsion, ultrasonication, optimization, Box–Behnken design, diabetes mellitus

## Abstract

The current study was intended to fabricate and evaluate ultrasonically assisted quercetin nanoemulsion (Que-NE) for improved bioavailability and therapeutic effectiveness against diabetes mellitus in rats. Ethyl oleate, Tween 20, and Labrasol were chosen as oil, surfactant, and cosurfactant, respectively. Box–Behnken design (BBD) was employed to study the influence of process variables such as % surfactant and cosurfactant mixture (Smix) (5 to 7%), % amplitude (20–30%) and sonication time (2.5–7.5 min) on droplet size, polydispersibility index (PDI), and % entrapment efficiency (%EE) were studied. The optimization predicted that 9% Smix at 25% amplitude for 2.5 min would produce Que-NE with a droplet size of 125.51 nm, 0.215 PDI, and 87.04% EE. Moreover, the optimized Que-NE exhibited appreciable droplet size and PDI when stored at 5, 30, and 40 °C for 45 days. Also, the morphological characterization by transmission electron microscope (TEM) indicated the spherical shape of the optimized nanoemulsion. Furthermore, the Que-NE compared to pure quercetin exhibited superior release and enhanced oral bioavailability. The streptozocin-induced antidiabetic study in rats revealed that the Que-NE had remarkable protective and therapeutic properties in managing body weight, blood glucose level, lipid profile, and tissue injury markers, alongside the structure of pancreatic β-cells and hepatocytes being protected. Thus, the developed Que-NE could be of potential use as a substitute strategy for diabetes.

## 1. Introduction

Quercetin (Que) is a natural bioflavonoid found in commonly consumed fresh green leafy vegetables and fruits. In recent times, Que has gained considerable interest due to its potential health benefits beyond antioxidant activity, including anti-inflammatory, anticancer, cardioprotective, antihypertensive, vasodilator effects, antiobesity, anti-hypercholesteraemic, neuroprotective, and antidiabetic properties [1]. Furthermore, in vivo studies have reported that Que at doses ranging from 15 mg/kg body weight (BW) to 100 mg/kg BW for 14 to 70 days exhibited a prominent antidiabetic effect [2]. However, the low aqueous solubility and poor oral bioavailability (~17%) have constrained its therapeutic efficacy and application [2,3]. Hence, a suitable delivery system for Que could overcome these pharmaceutical challenges and improve its oral bioavailability and therapeutic efficacy. In such efforts, the lipid-based delivery system is the right choice for delivering hydrophobic bioactive such as Que, as these systems increase the solubility and bioavailability of hydrophobic components [4,5,6].

Nanoemulsion is a lipid-based delivery system defined as a colloidal dispersion of two nonmiscible fluids such as oil and water, in which one forms the dispersed phase and the other forms a dispersion medium [7]. Though nanoemulsions are thermodynamically unstable, their smaller droplet size of <200 nm helps resist gravitational separation and droplet aggregation due to less attractive forces between the smaller droplets, hence making the system kinetically stable [7,8,9]. The smaller droplet size of the nanoemulsion also influences the rheological and release behavior of the components. Nanoemulsions require less surfactant preparation and are least affected by physical and chemical variations, including temperature and pH [8]. In addition to the solubility and bioavailability improvement, the nanoemulsion improves drug encapsulation efficiency compared to its counterparts [4]. These advantages of nanoemulsion can fulfill the quest for an efficient delivery system for Que [10,11] Nanoemulsions can be successfully developed using low-energy methods (LEMs) and high energy methods (HEMs). The advantage of adopting an HEM is that it requires less surfactant and is more suitable for oral administration. An HEM consists of ultrasonication, high-pressure homogenization, and microfluidization techniques. Frequent attempts have been made to develop a quercetin nanoemulsion and evaluate its various pharmacological benefits. Most reported studies have employed either high-pressure homogenization or spontaneous emulsification methods. However, the application of the ultrasonication technique to produce quercetin nanoemulsion was merely explored systematically [12,13,14,15,16]. The benefits of ultrasonication are that it requires a relatively lower amount of surfactant than other HEM methods, and produces a nanoemulsion with smaller droplet size and a PDI with prolonged stability [17].

Fabrication of a stable nanoemulsion using ultrasonication depends on various parameters, including ultrasonication time, percentage amplitude, oil-to-surfactant ratio (Smix), and temperature. Hence, it becomes essential to optimize these parameters to achieve a nanoemulsion with good stability. Modern mathematical tools such as response surface methodology (RSM) are beneficial over traditional optimization techniques in reducing the optimization difficulties of cost and time.

RSM is a statistical and mathematical approach that utilizes a polynomial model to explain the relationship between response and independent variables by employing multivariate models. Under RSM, the optimum processing condition can be obtained using either three-level full factorial design, central composite design (CCD), Box–Behnken design (BBD), or a Doehlert matrix (DM). However, BBD is a multivariant optimization technique based on a three-level incomplete factorial design. The BBD is advantageous over the other RSM methods due to its ability to predict optimized conditions with fewer experimental runs by avoiding the experiential runs outside the feasible operating conditions. There are many factors, including optimum ultrasonic power, percentage Smix, and sonication time to consider when optimizing an emulsion-based system using ultrasonication to yield an optimal droplet size, polydispersibility index, and zeta potential.

The components and their combinations play a vital role in developing stable and efficient nanoemulsions. Moreover, many studies also implied that BBD is a beneficial method for nanoemulsion optimization [18,19,20]. Thus, in the present study, BBD was used to study the influence of selected independent variables (Smix, % amplitude, and sonication time) on response variables (droplet size, PDI, and %EE). The significance of this study was the evaluation of the combination of ethyl oleate, Tween 20, and Labrasol to procure a stable nanoemulsion using an ultrasonication technique for the improved bioavailability and antidiabetic properties of Que.

Hence, the present study was designed to optimize the ultrasonically assisted Que-NE using BBD and evaluate the optimized Que-NE for improved oral bioavailability and antidiabetic activity using an in vivo animal model.

## 2. Materials and Methods

### 2.1. Materials Employed

The quercetin (Que) extra pure (99%) (CAS-6151-25-3) (Supplementary Figure S1) was obtained from Sisco Research Laboratories, Hyderabad, India. The ethyl oleate (98–103%) (CAS-9005-62-6) and Tween 20 (extra pure) (CAS-9005-64-5) were procured from Loba Chemie Pvt. Ltd., Mumbai, India. The Labrasol was gifted from Gattefosse, India. The orthophosphoric acid (HPLC grade—88%) (CAS-7664-38-2), acetonitrile (HPLC grade—99.9%) (CAS-75-05-8), and methanol (HPLC grade—99.9%) (CAS-67-56-1) were obtained from Merck Specialties Pvt Ltd., Mumbai, India. The streptozocin (STZ) (CAS 18883-66-4) was purchased from Sigma-Aldrich, Bangalore, India. Water employed was Millipore-filtered through a Milli-Q filtration system. Furthermore, all the other chemicals, reagents, solvents used in the present study were of analytical grade.

### 2.2. Solubility Assessment of Que

Que was added in a surplus amount to each of the centrifuge tubes containing 2 mL of different oils, including coconut oil (CO), gingelly oil (GO), castor oil (CAO), olive oil (OA), ethyl oleate (EO), corn oil (COO), and Labrafac PG (LAF). Surfactants including Tween 20 (T20), Tween 40 (T40), Tween 60(T60), Tween 80 (T80), Lauroglycol (LGL), Labrasol (LAB), and Labrafil (LFIL). Cosurfactants including isopropyl alcohol (IPA), propylene glycol (PG), and Transcutol P (TP) were mixed vigorously using a vortex mixer. The samples were then placed in an isothermal shaker for 72 h at 25 ± 02 °C. The samples after equilibration were centrifuged at 6000 rpm (REMI, Ultracentrifuge) for 10 min. The supernatant was collected and clarified through a 0.22 μm membrane filter. The filtrate concentration was measured spectrophotometrically at 361 nm utilizing a UV-1700 spectrophotometer (Shimadzu, Canby, OR, USA) [21].

### 2.3. Screening of Surfactants

Oil emulsification by the surfactant was also crucial for a stable emulsion. Hence, the T20, LAB, and LAF surfactants in which the drug had high solubility were screened for EO (oil) emulsifying ability. In water, 2.5 mL of 15 wt % surfactant solution was prepared, and 4 μL of oil was supplemented with brisk vortexing. The addition of the oil was continued until the mixture turned cloudy [21,22].

### 2.4. Screening of Cosurfactants

The various solubilizers as cosurfactants, involving IPA, PG, TP, and LAB, were selected at a fixed ratio (1:1) with T20 and inspected for their ability to produce a transparent nanoemulsion region by constructing pseudoternary phase diagrams (TPDs). The oil and Smix were evaluated in 15 different combinations (1:9, 2:8, 3:7, 4:6, 5:5, 6:4, 7:3, 8:2, 9:1, 1:2, 1:3, 1:5, 1:6, 1:7, and 1:8) to incorporate maximum possible ratios to define the frontiers of phases precisely in the phase diagrams [23].

### 2.5. Effect of Surfactant and Cosurfactant (Smix) Mass Ratio

The surfactant (T20) was blended with the cosurfactant (LAB) in the weight ratios of 1:0, 1:1, 1:2, 1:3, 1:4, 1:5, 5:1, 4:1, 3:1, and 2:1. For a detailed study of the phase diagrams, the Smix ratios were initially chosen with an increasing surfactant concentration, while the cosurfactant concentration was kept constant. Later, the surfactant concentration was kept constant with an increasing concentration of the cosurfactant. TPDs were constructed using the aqueous titration method, which involved step-by-step addition of water to each weight ratio of oil and surfactants, and then blending the components using a vortex mixer at 25 °C [24]. Based on visual observation, the clear, easily flowable, and transparent formulation was identified as the nanoemulsion phase. Fifteen distinct permutations of different weight ratios of oil and Smix (1:9, 2:8, 3:7, 4:6, 5:5, 6:4, 7:3, 8:2, 9:1, 1:2, 1:3, 1:5, 1:6, 1:7, and 1:8) were taken. The three axes of the phase diagram corresponded to the three components of the nanoemulsion system: aqueous, oil (EO), and Smix (T20:LAB) phases.

### 2.6. Preparation of Nanoemulsion

A measured quantity of Que was dissolved by vortexing in an organic phase constituting a specified amount of EO, T20, and LAB. The organic-phase components and dissolved Que were transferred to the aqueous phase and subjected to high-shear homogenization (T-25 Ultraturrax, IKEA, Staufen, Germany) at 5000 rpm for 5 min to form a crude emulsion. This emulsion was then subjected to ultrasonication (Vibra cell VCS 750–220, Sonics, with a power output ranging between 20% and 40% amplitude) at a predetermined amplitude and time.

### 2.7. Optimization of Nanoemulsion by Box–Behnken Design (BBD)

BBD is beneficial, as it contains combinations for which all factors are concurrently at their highest and lowest levels. Furthermore, the suggested runs help avoid experiments performed under extreme conditions for which undesirable results might occur, and hence provides a study design with fewer study runs, making it economical. BBD also was successfully employed to optimize an NE system previously [18]. A three-factor, three-level Box–Behnken experimental design was adopted to optimize the NE formulation in the present study. Design Expert^®^ (Version 10.0 Stat-Ease Inc., Minneapolis, MN, USA) was used to investigate the quadratic response surfaces represented by the second-order polynomial model. An Smix ratio (A) from 5 to 7% was selected while considering the amount of Smix required to emulsify EO, % amplitude (B) from 20–30%, and sonication time (C) from 2.5 to 7.5 min as independent variables, considering the feasibility and previously reported studies; whereas the droplet size, PDI, and %EE were the dependent variables [18].

### 2.8. Determination of Droplet Size, PDI, and Zeta Potential

The Que-NE’s particle size, PDI, and zeta potential were determined using a dynamic light-scattering technique with a Zetasizer ZS90 (Malvern, Worcestershire, UK). The NE samples were measured at 25 °C after diluting them with distilled water at a ratio of 1:100.

### 2.9. Determination of Entrapment Efficiency (% EE)

The % EE was defined as the amount of drug entrapped within the matrix core formed by the organic phase components. The Que-loaded NE was ultracentrifuged (REMI Electrotechnik Ltd., Vasai, India) at 12,000 rpm for 20 min, and the supernatant was collected and filtered through a 0.22 µm membrane filter. The Que was quantified using HPLC (Shimadzu HPLC system-LC20C, I prominence series) consisting of an autosampler system and quaternary pump equipped with a UV–visible detector. The Lab-solution was used to process the chromatograms. The Que was eluted effectively by using a C18 column (Shimadzu, 5 µm particle size ODS, 150 mm × 4.6 mm) set at a temperature of 30 ± 2 °C. The injection volume was 20 µL, and the mobile phase comprised methanol and 0.1% phosphoric acid (98:2, *v/v*) at a flow rate of 1 mL/min; the effluent was detected at 361 nm [25]. The encapsulation efficiency was calculated and expressed in percentage [26].

### 2.10. Effect of Storage Temperature on Droplet Size and PDI

The Que-NE was prepared and transferred into glass vials and stored in the dark at 5, 30, and 40 °C for 45 days. The droplet size and PDI were measured accordingly on days 0, 7, 15, 30, and 45.

### 2.11. Morphological Characterization by Transmission Electron Microscope (TEM)

Transmission electron microscopy (TEM) was employed for morphological analysis and globular-size confirmation (JEOL/JEM 2100, Tokyo, Japan). A sequence of bright field imaging at increasing magnification and diffraction modes was applied to reveal the form and size of the nanoemulsion. The sample was prepared by diluting the optimized Que-NE in distilled water (1:100), and a drop of the sample was positioned on a copper grid and stained with uranyl acetate for 30 s. The stained grid was dried, placed on a slide, covered with a coverslip, and examined under the microscope [27].

### 2.12. Animals

Albino Wistar rats of about 200–250 g in weight were procured from Biogen Laboratory Animal Facility (Bengaluru, India). The rats were acclimatized under a controlled housing environment of 12 h/12 h light/dark cycles at a temperature of 23 °C for 15 days with free access to feed and water.

### 2.13. In Vivo Pharmacokinetics Study

Healthy male Albino Wistar rats weighing 250–300 g were purchased and acclimatized at 23 ± 2 °C at 50 ± 5% humidity and in a 12 h/12 h light/dark cycle condition for seven days. The housed rats had free access to standardized mouse pellet feed and potable water.

After acclimation, 12 rats were arbitrarily allocated into two groups (*n* = 6). All the rats were fasted overnight with access only to water before the experiments. Group A animals were administered 50 mg/kg of Quercetin pure drug (Que-PD), and Group B animals were administered 50 mg/kg of optimized Que-NE as a single oral dose through oral gavage. The blood samples (300 µL) were collected from the tail vein into a centrifuge tube containing 100 µL of 3% sodium citrate solution at 0 h (before dosing), 1 h, 2 h, 4 h, 8 h, 16 h, 24 h, and 48 h after oral administration. The collected blood samples were centrifuged immediately at 8000 rpm for 10 min. The plasma was collected and stored at −80 °C until further analysis by HPLC. The pharmacokinetic parameters were computed by the noncompartmental model using Pumas software (Julia Computing, Newton, MA, USA) [28].

### 2.14. In Vivo Antidiabetic Study

#### 2.14.1. Induction of Diabetes and Animal Grouping

The acclimatized healthy male Albino Wistar rats weighing 250–300 g were involved in this study. The streptozocin (STZ) was reported to effectively induce diabetes in rats in a single dose, making it a comparatively economical and faster method for induction of diabetes. However, STZ does not develop insulin resistance; the antidiabetic activity concerning the bodyweight management, blood glucose level, serum lipid levels, oxidative stress, and tissue injury markers, along with the pathological changes of β cell derangement due to diabetic glucotoxicity, could be well established [29]. Thus, STZ-induced diabetes in the rat model was employed in this study. The STZ injection was prepared by dissolving STZ in a citrate buffer at 4.5 pH. Overnight-fasted animals were injected with STZ (40 mg/kg body weight) intraperitoneally for type I diabetes induction. After 72 h of STZ injection, the blood glucose level from nonfasted rats was determined, and rats with >250 mg/dL were considered to have developed type I diabetes. Furthermore, 30 animals were allotted into five groups (*n* = 6): Group A-Normal (without any induction), Group B-Control (STZ-induced diabetes), Group C-Standard (diabetes-induced and metformin-treated), Group D-Que-NE (diabetes + Que-NE-treated), and Group E-Que-NE (P) (Que-NE pretreatment + diabetes). Groups A and B received 1.5 mL of distilled water daily, whereas Group C received metformin at 250 mg/kg BW daily, Group D received 12.5 mg/kg BW of Que-NE, and Group E received 12.5 mg/kg of Que-NE (from seven days before induction of type I diabetes until the end of the experiment). All doses were administered orally for 21 days, with close observation of the animals during the experimental period.

After 21 days of treatment, the blood sample was collected by the retro-orbital plexus method after the animals were euthanized. The blood collection, storage, and measurements were performed as described previously [30]. Serum was isolated by centrifugation at 8000 rpm for 15 min. A Varioskan LUX Multimode Microplate Reader (Thermo Fisher Scientific, Waltham, MA, USA) was used for the analysis. Serum alanine aminotransferase (ALT), aspartate aminotransferase (AST), total cholesterol (TC), total glycerides (TG), high-density lipoproteins (HDL), and low-density lipoproteins (LDL) were determined using the kit method (Spinreact, Girona, Spain). Creatinine (Cre) and blood urea nitrogen (BUN) levels were measured using kits from Elabscience, (US). VLDL was calculated as described earlier [31].

The liver was removed, washed thrice in ice-cold saline, and blotted separately, then tissue homogenates were prepared to estimate tissue malondialdehyde (MDA) and superoxide dismutase (SOD) levels using a previously established procedure [32].

#### 2.14.2. Measurement of Blood Glucose Level (BGL), Food Intake, and Water Consumption

The BGLs of all the rats were measured at 0, 7, 14, and 21 days through the tail vein method using a blood glucose meter (ACCU-CHEK) that employed test strips to determine the BGL, according to the manufacturer’s instructions. The STZ-induced hyperglycemic condition resulted in extreme thirst (polydipsia) and hunger (polyphagia). Thus, the average food and water intake was calculated on a daily basis by calculating the difference in food and water provided and consumed per cage [33].

#### 2.14.3. Measurement of Bodyweight (BW)

The animals’ bodyweights were measured using a calibrated weighing machine at 0, 7, 14, and 21 days.

#### 2.14.4. Oral Glucose Tolerance Test (OGTT)

The animals were administered glucose (2 mg/kg) after overnight fasting for 12 h on the 10th and 20th days during the study. The blood glucose level was monitored at 0, 20, 60, 90, and 120 min after glucose administration [30,32].

#### 2.14.5. Histopathological Analysis of Pancreas and Liver Tissues

The liver and pancreatic tissue samples were dissected, rinsed, and fixed using 10% formalin solution. Then, the samples were embedded in paraffin, and sections were made and stained using hematoxylin and eosin and subjected to microscopic examination.

## 3. Results and Discussion

### 3.1. Screening of Nanoemulsion Components

**Figure 1:**
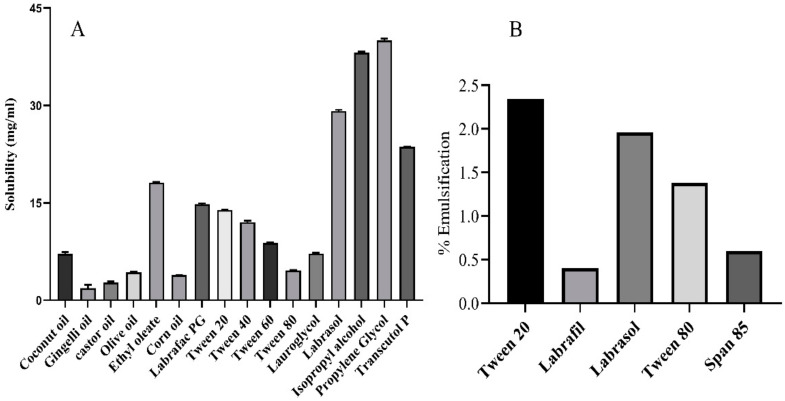
(**A**) Quercetin solubility in various nanoemulsion components; (**B**) ethyl oleate emulsification by selected surfactants.

Quercetin’s solubility in different nanoemulsion components such as oils, surfactants, and cosurfactants was determined, as shown in Figure 1A. Quercetin showed a higher solubility in EO (18.12 ± 0.10 mg/mL) than in other oils. The solubility of the drug in the oily phase was an essential feature in developing a nanoemulsion formulation, because drug loading (the ability of the preparation to keep the drug in the solubilized form in GI) and volume of preparation for delivery of the therapeutic dose greatly depended on its solubility in the oil phase [21]. Therefore, selecting a suitable surfactant was crucial, as it could induce gastrointestinal toxicity when the formulation was administered orally [21]. Hence, nonionic surfactants were preferred in this study due to their reduced toxicity, ability to withstand pH, ionic conditions, lower CMC value, and, most importantly, biocompatibility [21]. In addition, nonionic surfactants in a nanoemulsion deliver superior in vivo stability [31]. Therefore, considerations were made to screen surfactants in a wide range of HLB values (10–16.7), as the value required to form an *o/w* nanoemulsion was higher than 10. In addition, hydrophilic surfactants required lower energy to form nanoemulsions and, consequently, improve the formulation’s stability [21]. Hence, in the present study, nonionic surfactants were assessed primarily for their ability to emulsify the nanoemulsion system, rather than the solubility of the drug quercetin alone. Among a series of anion surfactants (T20, T40, T60, T80, LGL, and LAB), T20-emulsified EO fared better, as depicted in Figure 1B; this could be attributed to the higher HLB value of T20. Hence, T20 was selected as the primary surfactant.

**Figure 2:**
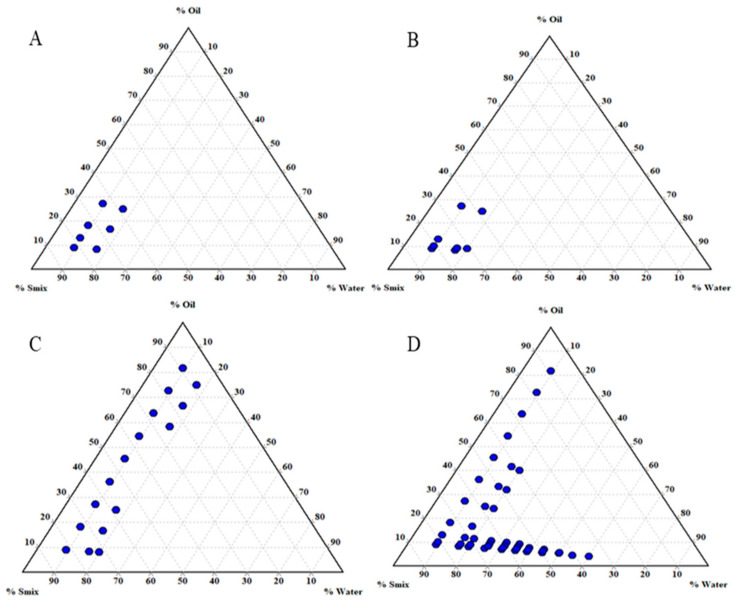
Ternary phase diagrams of nanoemulsions consisting of ethyl oleate and Tween 20 with different cosurfactants: (**A**) isopropyl alcohol; (**B**) propylene glycol; (**C**) Transcutol P; (**D**) Labrasol at a 1:1 ratio.

The cosurfactant was selected to reduce the interfacial tension and produce a larger NE region in the constructed phase diagrams. Cosurfactants such as IPA, PG, TP, and LAB were screened for producing a larger NE region with the T20 surfactant at a 1:1 ratio. It was observed that the TP could increase the NE region compared to that of IPA and PG. However, LAB could significantly increase the NE region compared with the other cosurfactants (TP, IPA, and PG), as represented in Figure 2. LAB as a medium-chain triglyceride (MCT) is also known to improve lipophilic drugs’ bioavailability and GI permeation [32,34]. Moreover, the NE system of Que with Tween 20 and Labrasol as a surfactant mixture and ethyl oleate as oil phase had not been discovered to date. Hence, these components were selected for further assessment.

### 3.2. Effect of Smix Mass Ratio

An NE’s formation is chiefly based on its composition and its order of addition [35]. Therefore, TPD was used to represent the NE region. In the present study, the phase diagram was constructed with EO, T20, and LAB as the oil, surfactant, and cosurfactant, respectively. The T20 and LAB were added to the EO and were titrated against the aqueous phase. The obtained phase diagrams were evaluated in comparison to previous studies, and it was considered that the transparent region obtained in the phase diagrams indicated the nanoemulsion region [36]. Furthermore, the other region exhibiting turbidity was considered conventional emulsion systems. No formulation showed specific conversion from an *o/w* to *w/o* NE.

**Figure 3:**
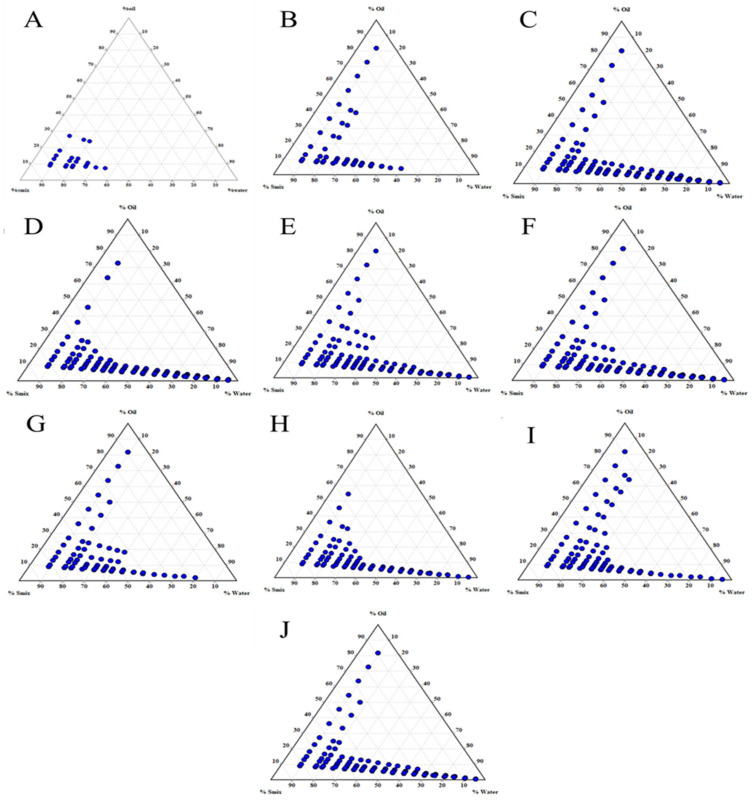
TPDs of nanoemulsions consisting of ethyl oleate and Smix (Tween 20:Labrasol) at Smix ratios of: (**A**) 1:0; (**B**) 1:1; (**C**) 2:1; (**D**) 3:1; (**E**) 4:1; (**F**) 5:1; (**G**) 1:2; (**H**) 1:3; (**I**) 1:4; (**J**) 1:5.

**Figure 4:**
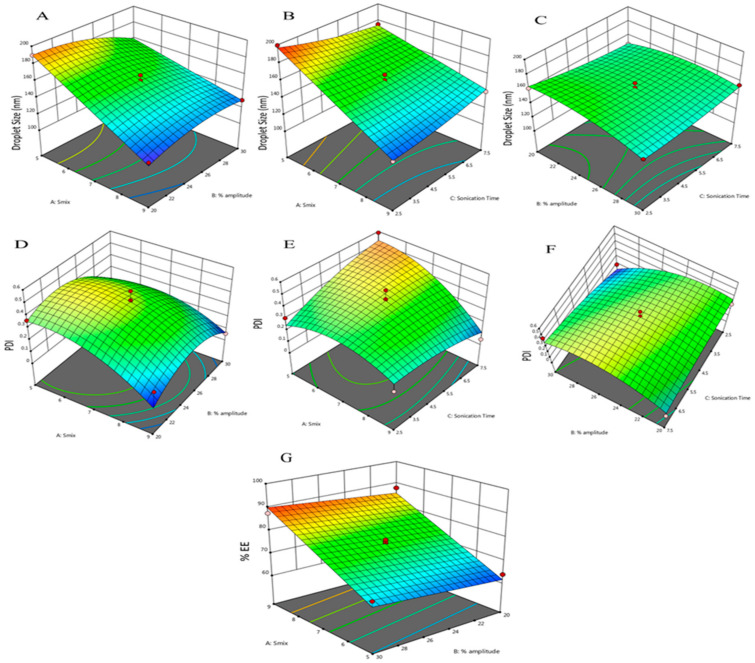
3D Response surface plots representing the interaction effect for the droplet size of Que-NE (**A**) Smix and % amplitude, (**B**) Smix and sonication time, and (**C**) % amplitude and sonication time; the interaction effect for PDI of Que-NE (**D**) Smix and % amplitude, (**E**) Smix and sonication time, and (**F**) % amplitude and sonication time; and the interaction effect for %EE of Que-NE (**G**) Smix and % amplitude.

The effect of the Smix mass ratio was assessed further to optimize the NE system (Figure 3). A lower NE region was noted (Figure 3A), in which the T20 surfactant alone (Smix 1:0) was employed, indicating that T20 alone could not reduce the interfacial tension between the oil and water phase, and addition of the cosurfactant was essential. An increase in the NE area was observed with an Smix ratio of 1:1; i.e., in the presence of the LAB cosurfactant (Figure 3B). Increasing the surfactant concentration further; i.e., at Smix 2:1, led to an increase in the NE region (Figure 3C). Smix ratios of 3:1 (Figure 3D) and 4:1 (Figure 3E) also led to a corresponding increase in the NE region, which gradually began decreasing at a Smix ratio of 5:1, signifying that an optimum emulsion was achieved at Smix 4:1. Correspondingly, when the concentration of cosurfactant was increased, while keeping the surfactant concentration constant at 1:2 (Figure 3G), an increase in the NE region was observed compared to Smix at a 1:1 ratio. When the Smix ratio was further increased to 1:3, a slight decrease in the NE region was observed. Correspondingly, an increase in the NE region was seen at Smix 1:4. However, a further increase in the Smix ratio up to 1:5 did not improve the NE region. Primarily, this could be correlated with the HLB value of the Smix combinations. The T20 had an HLB of 16, and the LAB of 12. Overall, the HLB value of the Smix ranged from 14.00 to 16.00 upon an increment in the surfactant concentration, and ranged from 12.80 to 14.00 upon an increment in the cosurfactant concentration. The highest NE region was formed at Smix 1:4 (Figure 3I), indicating that an HLB value of 12.80 would be required to achieve optimum emulsification of EO and produce NE. Secondly, the higher viscosity of T20 could have decreased in the NE region, as high viscosity would negatively affect droplet disruption and result in the breaking of the emulsion. In addition, the NE developed using a phase diagram showing the nanoemulsion region toward the water-rich apex could be diluted to a greater degree [21]. An Smix concentration between 3 and 10% allowed nanoemulsion formation [37]. Hence, in the present study, the concentration of the entire organic phase (including oil, surfactant, and cosurfactant) was maintained at 10% of the total formulation, wherein the oil phase ratio was constant at 1, and the Smix ratio was varied from 5 to 9.

### 3.3. Optimization of Nanoemulsion by BBD

The experimental array for droplet size (nm), PDI, and entrapment efficiency (%) was 120.9 ± 10.0 to 199.5 ± 27.68 nm, 0.056 ± 0.04 to 0.59 ± 0.03, and 60.16 ± 5.19 to 91.08 ± 2.95%, respectively (Table 1). When the achieved response values were studied using design experiment software, the quadratic polynomial model was suggested for droplet size (*p* < 0.05) and PDI (*p* > 0.05), and the linear model was suggested for entrapment efficiency (*p* < 0.001). The ANOVA analysis, fit statistics, and regression coefficient values are shown in Table 2, and the regression equations obtained are summarized in Table 3. It was found that the lack of fit was insignificant (*p* > 0.05) for all the responses. The larger *F*-value and smaller *p*-value confirmed the significant effect of each variable. (Table 2).

The obtained data were used for the calculation of coefficients of the quadratic polynomial equation. Regression equations for different responses obtained from design expert software are represented in Table 2. The ANOVA results exhibited that the quadratic polynomial model could signify experimental data, with the values of the coefficient of determination (R^2^) of particle size, PDI, and EE of Que-NE being 0.9599, 0.8191, and 0.9397, respectively, as described in Table 2.

The droplet size of Que-NE was primarily dependent on the linear effect of the Smix ratio (*p* < 0.001), the interaction effect among Smix and % amplitude (*p* < 0.05), and the quadratic effect of % amplitude (*p* < 0.05). Thus, an increased Smix ratio reduced droplet size with a correspondingly higher amplitude due to increased shear and cavitation forces [36]. The other variables that had a significant effect on droplet size were the linear effect of % amplitude (*p* < 0.05) and the interaction effect between the Smix and sonication time (*p* < 0.05). The interactive effect of the Smix and % amplitude are depicted in Figure 4A. These variables exhibited a linear effect on the droplet size; i.e., the droplet size reduced at a higher % amplitude due to the generation of shear forces, which broke the larger droplets into smaller ones [38]. In addition, a higher Smix ratio reduced the system’s interfacial tension, resulting in a smaller droplet size [39].

The interaction effect of Smix and sonication time are represented in Figure 4B. Both the variables were found to exhibit a linear effect on the droplet size. As the Smix and sonication time were increased, the Que-NE droplet size was found to decrease. It was reported that when NE was subjected to high shear for a prolonged duration, it reduced the larger droplet into a smaller one [38]. The interactive effect of % amplitude and sonication time is depicted in Figure 4C, and was not found to affect the droplet size (*p* > 0.05).

The PDI of Que-NE was primarily dependent on the linear effect of Smix, the interaction effect of % amplitude, sonication time, and the quadratic effect of % amplitude. The interaction effect of Smix, % amplitude, and sonication time on PDI are depicted in Figure 4 D–F. It could be seen that at lower Smix and % amplitude, the PDI was high; however, with an increase in Smix and % amplitude, it was reduced. Sonication time and % amplitude had a prominent effect on PDI due to high shear, resulting in homogenous droplet size in the nanoemulsion [40].

The % EE was primarily affected by linear terms of Smix (*p* < 0.001) and % amplitude (*p* < 0.05). Furthermore, no interactive or quadratic model was found to have significant effects. The linear effect of Smix and % amplitude is depicted in Figure 4G. The quadratic equations obtained (Table 3) suggested that a higher Smix with an optimum % amplitude for a shorter sonication time would yield a smaller droplet with an optimum PDI and a higher entrapment efficiency.

The model was further verified by formulating the Que-Ne under obtained optimum conditions. The experimental data were found to be in good agreement with the predicted values (Table 4). Thus, we concluded that BBD could be successfully employed to study the linear, interactive, and quadratic effects of Smix, % amplitude, and sonication time on NE droplet size, PDI, and %EE. Therefore, BBD could be successfully applied to optimize the Que-NE with the desired responses. The zeta potential of the optimized formulation was −17.10 ± 5.61 mV. The negative zeta potential could be attributed to the dissociation of fatty acid adsorbed or the presence of negatively charged ions at the interface. In the present study, the negative zeta potential could be due to the hydroxyl group in the quercetin and ethyl oleate that were employed [41,42].

### 3.4. Effect of Storage Conditions on Droplet Size and %EE

**Figure 5:**
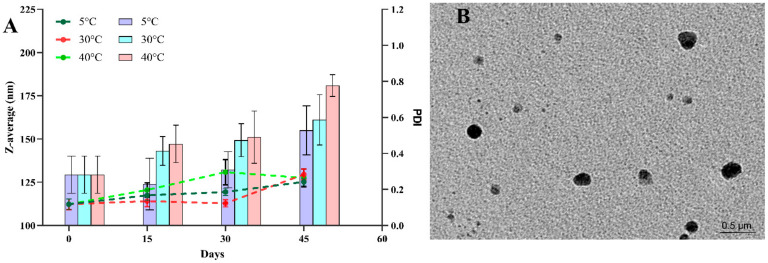
(**A**) Effect of storage temperature on droplet size (Z-average (nm)) and PDI of Que-NE at 5, 30, and 40 °C for 45 days; (**B**) TEM image of optimized Que-NE.

An NE’s stability is an essential parameter for its application. The droplet size and the PDI are prominent indicators of NE stability. In the present study, stability testing of optimized Que-NE was examined at 5, 30, and 40 °C on days 0, 15, 30, and 45. As depicted in Figure 5A, the droplet sizes tended to increase during days 0–15 in all storage conditions except at 5 °C, at which it decreased. However, it was found to increase beyond the 15th day. The droplet size of the emulsion stored at 40 °C demonstrated an increasing trend at the 45th day due to the coalescence of NE droplets. There was no visible appearance of creaming or phase separation until the 45th day of the study. The droplet size and PDI of Que-NE stored at 30 °C was the least affected after 45 days of storage. All the NEs exhibited a PDI ≤ 0.3, and were considered to have good stability under the tested conditions.

### 3.5. Morphological Characterization by Transmission Electron Microscopy (TEM)

The TEM micrographs of Que-NE indicated that the droplets were spherical and in good agreement with the droplet size results, confirming the feasibility of the optimized Que-NE formulation. The microdroplets appeared dark, and the surroundings were bright (Figure 5B).

### 3.6. In Vivo Pharmacokinetic Studies

**Figure 6:**
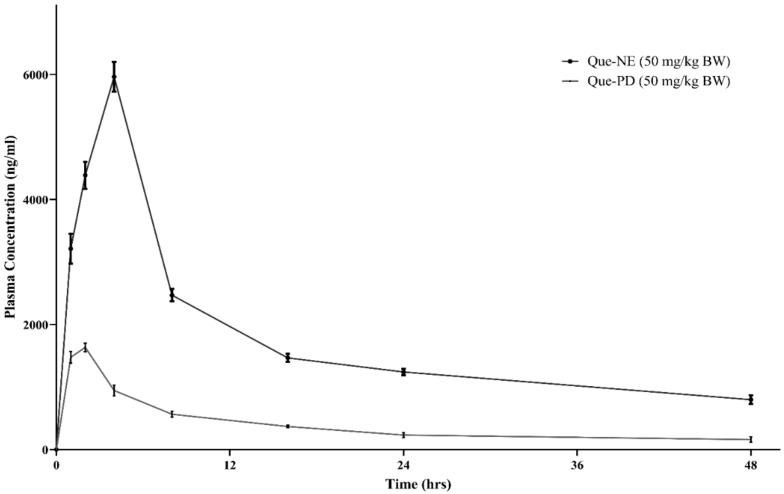
Plasma concentration–time profile of optimized Que-NE and Que-PD.

The HPLC technique was employed to study the pharmacokinetics of Que-PD and Que-NE in rat plasma after administration of a single oral dose. The mean plasma concentration–time profile is shown in Figure 6. The maximum plasma concentration (Cmax) and the time taken to reach Cmax (tmax) were obtained directly from this profile. The other critical pharmacokinetic parameters were calculated by the noncompartmental model, and are represented in Table 5. The Que-NE and Que-PD after a single oral administration reached the maximum drug concentration (Cmax) of 5962.74 ± 238.54 ng/mL and 1634.28 ± 70.18 ng/mL after 4 ± 0.0 and 2 ± 0.0, respectively. It was observed that in contrast to Que-PD, the Cmax of Que-NE was 3.64-fold greater, indicating that the NE system was effective in increasing the Que absorption. The Que-NE had a delayed tmax compared with that of Que-PD, indicating that the NE system could have resulted in the sustained release of Que from the system. Additionally, the mean resident times (MRTs) of Que-NE and Que-PD were 46.13 ± 9.91 h and 28.78 ± 8.44 h, respectively. This higher MRT could be attributed to the sustained release of Que into the systemic circulation. The NE system had decreased the distribution of Que by 3.04 times compared with that of Que-PD. In contrast to Que-PD, the AUC0-t and AUC^0^^−^^∞^ were 4.46 and 5.32 times higher, respectively. These indicated that Que-NE had enhanced oral bioavailability due to the drug’s absorption and residence time delivered from the NE system [43].

The improved oral bioavailability by the NE system could be attributed to the improved permeability by the Smix employed, reduced gastric degradation of Que, and reduced clearance. Initially, the absorption of Que in the NE form may have been well taken up through the GI tract, where the droplet size played a primary role in the absorption rate [44,45]. The smaller droplet size (<200 nm) of Que-NE allowed an effective uptake in the intestine, mainly in the lymphoid tissue, thus bypassing the first-pass metabolism [45]. Furthermore, the surfactants, such as T20 and LAB, had increased permeability or enhanced the affinity among lipid molecules and the intestinal membrane, thus showing bioadhesion to the GI tract wall [46]. Thirdly, by encapsulating into nanodroplets, Que could be embedded into a lipid matrix, thus not only reducing its exposure to the bacterium, as well as enzymatic degradation throughout absorption, but also contributing to prolonged contact with the wall of the intestine in vivo due to the potential adhesion of NE to the mucosal surface of intestinal tissue [47]. Moreover, this system could influence Que with a prolonged circulation in vivo in a sustained manner, extended the Que’s systematic residence time, and improved bioavailability [44].

**Figure 7:**
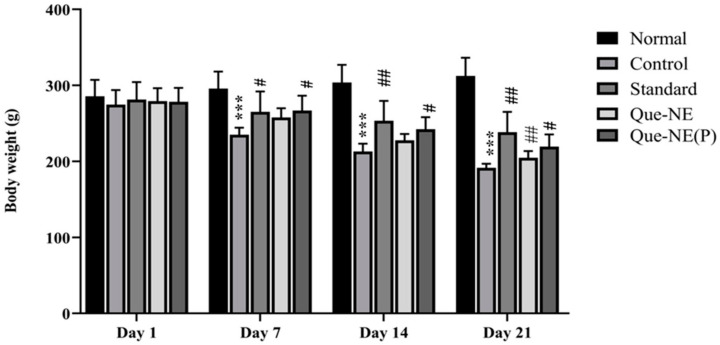
Effect of Que-NE and Que-NE (P) on body weight (BW) measured on days 1, 7, 14, and 21 of treatment. Data presented are the mean ± standard deviation. Significance was measured using one-way ANOVA. *** *p* < 0.001 vs. Normal group. # *p* < 0.05 and ## *p* < 0.01 vs. Control group (*n* = 6).

The effect of Que-NE on the BW of rats is depicted in Figure 7. It was observed that the Control group showed a significant loss in body weight on the 7th day when compared with that of the Normal group (*p* > 0.001). In contrast, the standard (*p* > 0.05) and Que-NE (P) (*p* > 0.05) treatment groups showed significantly inhibited weight loss. A similar trend was followed on the 14th day, with the Control group showing further weight reduction when compared with that of the Normal group. However, on the 21st day, the Que-NE and Que-NE (P) groups showed significant inhibition in bodyweight reduction in comparison with the Control group (*p* > 0.05). In addition, it was prominent that Que-NE (P) had improved bodyweight compared with that of Que-NE, signifying the protective effect of Que.

### 3.7. Effect of Que-NE and Que-NE (P) on Blood Glucose Level (BGL), Food Intake, and Water Consumption

**Figure 8:**
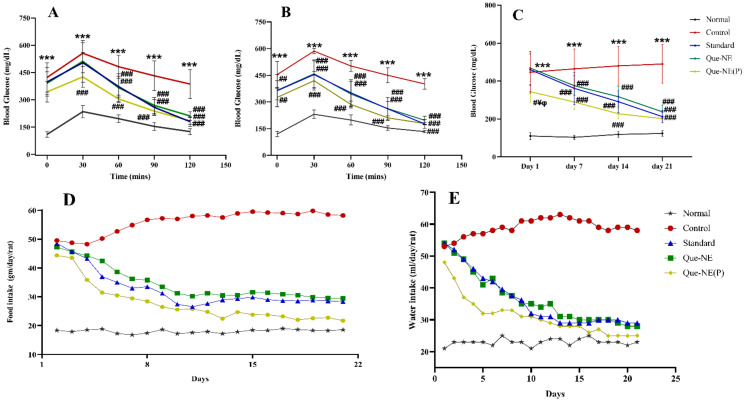
Effects of Que-NE and Que-NE (P) on: (**A**) oral glucose tolerance test on the 10th day; (**B**) oral glucose tolerance test on the 20th day; (**C**) blood glucose level (BGL); (**D**) food intake; (**E**) water intake. Data presented are the mean ± standard deviation. Significance was measured using one-way ANOVA: *** *p* < 0.001 vs. Normal group. # *p* < 0.05, ## *p* < 0.01, and ### *p* < 0.001 vs. Control group. ¥ *p* < 0.05 vs. Standard group, and ^φ^ *p* < 0.05 vs. Que-NE treatment (*n* = 6).

The influence of Que-NE on BGL is depicted in Figure 8C. The BGL gradually increased in the Control group compared with that of the Normal group (*p* > 0.05), while the standard, Que-NE, and Que-NE (P) groups showed significantly (*p* > 0.001) decreased blood glucose levels. In particular, the Que-NE (P) group showed a significantly decreased level from the 1st day onwards (*p* > 0.001). On the 7th day, the BGL of the Control group increased significantly (*p* > 0.001) over the Normal group, while the standard, Que-NE-, and Que-NE (P)-treated groups demonstrated a significantly decreased BGL compared with the controlled group until 14th day. On the 21st day, the BGLs of all the treated groups were closer to each other. It was notable that Que-NE (P) showed the lowest BGL among all the treated groups. Que has been reported to effectively lower the BGL by increasing insulin sensitivity, glycogen synthesis, and improving liver gluconeogenesis [27,48]. The average food and water intake are represented graphically in Figure 8D,E. The maximum feed and water intake of the Normal group was 18.90 gm/day/rat and 25.0 mL/day/rat, respectively. The Control group showed relatively higher consumption of food and water, reaching up to 59.80 gm/day/rat and 63.0 mL/day/rat, respectively. However, all the treatment groups, including standard, Que-NE, and Que-NE (P), showed an inhibition of the increase in food and water consumption remarkably. It was observed that the Que-NE had a similar therapeutic effect on food and water intake compared with that of standard. The Que-NE (P) had a prominent effect on food and water intake compared with all other treated groups due to the prior treatment, as it notably reduced the intake of food to 21.7 gm/day/rat and water intake to 25.0 mL/day/rat on the 21st day of the treatment. Hence, we concluded that Que had a potential effect on polydipsia and polyphagia, and the nanoemulsion had conceivably enhanced the therapeutic effect of Que due to increased bioavailability.

The OGTTs conducted on the 10th (Figure 8A) and 20th days (Figure 8B) also showed that the glucose tolerance was compromised in the Control group. However, all the other treated groups improved their tolerance. It was notable that Que-NE (P) showed better tolerance than Que-NE and standard, indicating the potential protective effect of Que. Furthermore, correlating the dose administered and BGL achieved, one could say that the enhanced bioavailability of Que through the nanoemulsion system would have enhanced the effectiveness of Que.

### 3.8. Effect of Que-NE and Que-NE (P) Treatment on Serum Lipid Level

**Figure 9:**
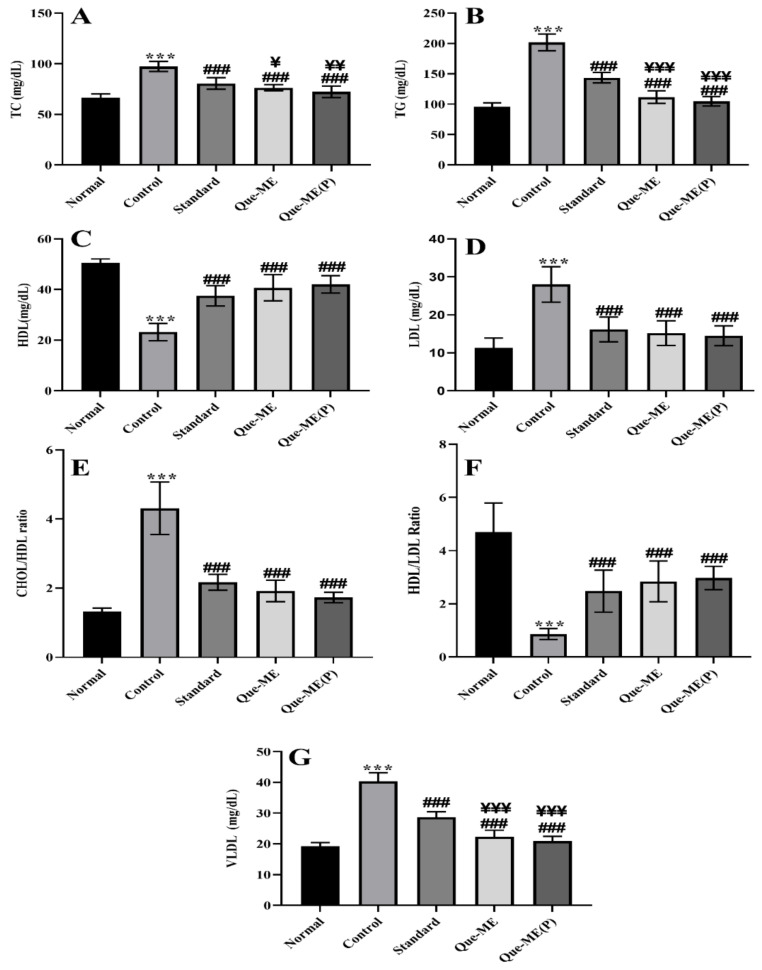
Effect of Que-NE and Que-NE (P) on various lipid profiles: (**A**) total cholesterol—TC; (**B**) total glycerides—TG; (**C**) high-density lipoproteins—HDL; (**D**) low-density lipoproteins—LDL; (**E**) cholesterol-to-HDL ratio—CHOL/HDL ratio; (**F**) HDL/LDL ratio; (**G**) very-low-density lipoproteins—VLDL. Data presented are the mean ± standard deviation (*n* = 6). Significance was measured using one-way ANOVA. *** *p* < 0.001 vs. Normal group. ### *p* < 0.001 vs. Control group. ¥ *p* < 0.05, ¥¥ *p* < 0.01, and ¥¥¥ *p* < 0.001 vs. Standard group (*n* = 6).

The serum lipid levels were measured to investigate the effect of the Que-NE system on lipid levels, and are represented in Figure 9. Total cholesterol (TC), total glycerides (TG), low-density lipoprotein (LDL), high-density lipoprotein (HDL), and very-low-density lipoprotein (VLDL) levels were significantly elevated in the Control group compared with the Normal group. In contrast, the treatment groups showed their levels to be near normal. The HDL level was found to be decreased in the Control group, whereas the standard, Que-NE-, and Que-NE (P)-treated groups showed a significant improvement in their levels. It has been established that dyslipidemia is the leading risk factor for various diabetes and other complications [49]. In the present study, Que-NE, especially in the Que-NE (P) group, had a prominent effect on lipid levels compared to Que-NE, indicating that pretreatment with Que could reduce or prevent various diabetic complications by inhibiting dyslipidemia. The obtained results can be correlated with the previous studies wherein a nanoemulsion-based delivery system effectively enhanced therapeutic efficacy by improving the stability and bioavailability of the bioactive compared with the conventional delivery system [50].

### 3.9. Effect of Que-NE and Que-NE (P) on Tissue Injury and Oxidative Stress Markers

**Figure 10:**
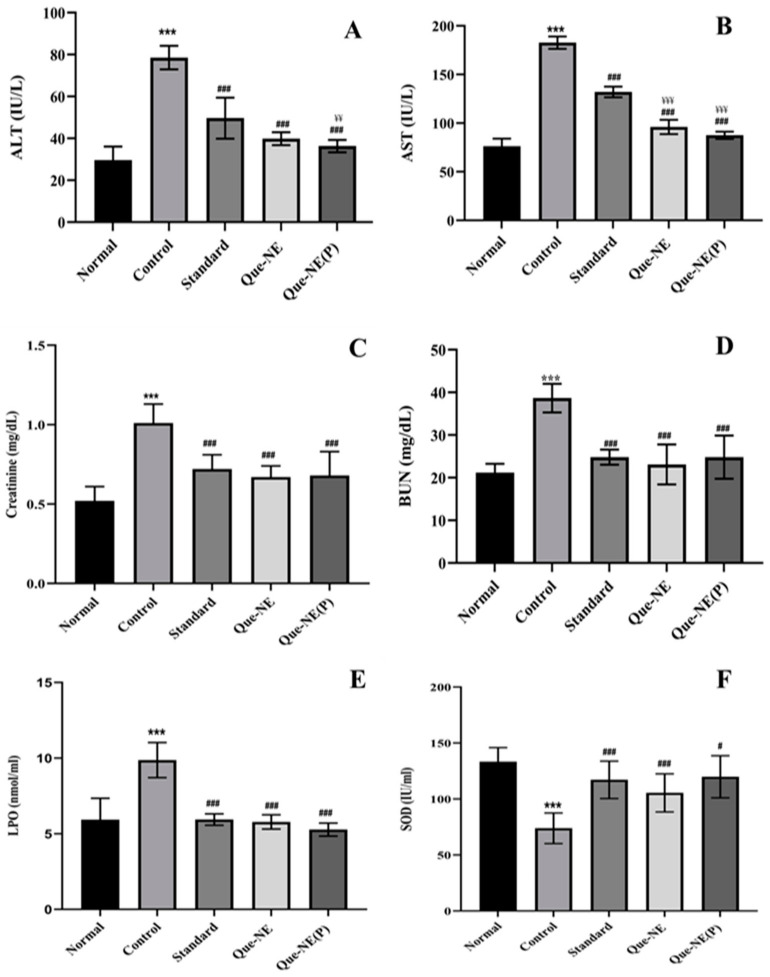
Effect of Que-NE and Que-NE (P) on: (**A**) alanine aminotransferase—ALT; (**B**) aspartate aminotransferase—AST; (**C**) creatinine; (**D**) blood–urea–nitrogen—BUN; (**E**) lipid peroxidation; (**F**) superoxide dismutase—SOD. Data presented are the mean ± standard deviation. Significance was measured using one-way ANOVA. *** *p* < 0.001 vs. Normal group. # *p* < 0.05, and ### *p* < 0.001 vs. Control group. ¥¥ *p* < 0.01, and ¥¥¥ *p* < 0.001 vs. Standard group (*n* = 6).

The AST and ALT are liver injury markers, while BUN and creatinine are the vital factors to identify the tissue injury. Higher levels imply the initiation of tissue damage due to diabetes-induced oxidative stress [51]. The hepatoprotective property of Que was attributed to its improved lipophagy [48]. Moreover, the nephroprotective property of Que was associated with inhibition of protein kinase C activity and downregulation of transforming growth factor β1 (TGF-β1) [52]. In this study, elevated levels of AST and ALT were seen in the Control group, whereas the treated groups demonstrated a significant reduction in the levels of these markers. Overall, Que-NE (P) showed slightly superior protection than the Que-NE, and proved that Que as nanoemulsion could enhance the potential to inhibit or prevent injury or diabetic complications (Figure 10).

### 3.10. Histopathological Analysis of Liver and Pancreatic Tissue

**Figure 11:**
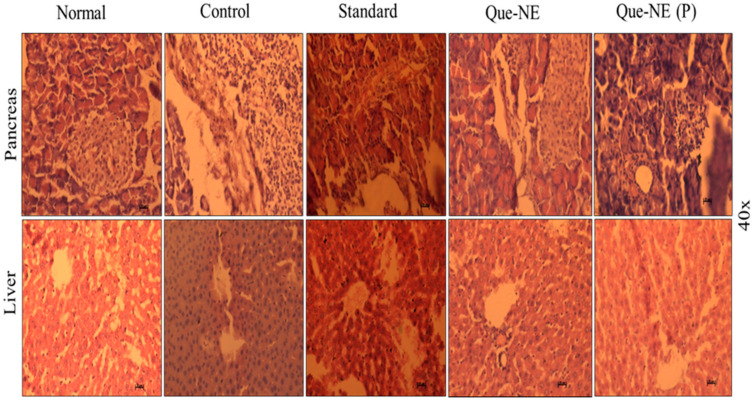
Histopathological observation of the effect of Que-NE and Que-NE (P) treatment on pancreatic and liver tissue after 21-day treatment (scale bar = 50 µm).

In the histopathological observations, the Control group exhibited disorganized islets of Langerhans and clusters of inflammatory cells. The β-cells showed dark-stained degenerated nuclei, and the liver portal vein was dilated with increased dark-stained hepatocytes due to apoptosis in the liver. A typical pancreatic structure with islets of Langerhans surrounded by exocrine acini and a natural population of β-cells was observed in the Normal group rats. The standard treated group exhibited an organized pancreas with decreased β-cells and darkly stained inflammatory cells. Sections of the liver cells showed a normalized liver with prominent hepatocytes. In the Que-NE treatment, the liver showed a typical structure, with well-organized portal veins with slight sinusoidal dilation, and the β-cells were observed to be elongated. However, the population of β-cells was normal with no degenerative changes. The Que-NE (P)-treated group showed reorganized pancreas cells with slight vacuolation in the Langerhans, and a normalized hepatic structure with radially arranged hepatocytes around the central vein was observed (Figure 11).

## 4. Conclusions

The present study fabricated a quercetin nanoemulsion using EO, T20, and Labrasol as oil, surfactant, and cosurfactant, respectively. The BBD was employed to study the influence of Smix, % amplitude, and sonication time on droplet size, PDI, and %EE of the nanoemulsion, and established the optimum conditions (9% Smix, 25% amplitude and 2.5 min of ultrasonication time). The optimized quercetin nanoemulsion exhibited good stability for 45 days. The Que-NE had superior oral bioavailability compared to Que-PD. Que at 12.5 mg/kg BW showed significant protective and therapeutic activity against STZ-induced diabetes for 21 days, controlled bodyweight and blood glucose level, and inhibited elevated serum lipid levels. In addition, it significantly inhibited tissue injury and oxidative stress markers. Therefore, we concluded that ultrasonically assisted Que-NE had improved oral bioavailability, and enhanced Que’s therapeutic and protective antidiabetic effect.

## Figures and Tables

**Table 1:** Box–Behnken design for Que-NE with independent variables and their response values (Supplementary Figure S2).

Run	Independent Variables			Response Values		
	S_mix_ * (%)	% Amplitude	Sonication Time (min)	Droplet Size (nm)	PDI	EE (%)
1	7	30	7.5	155.0±12.83	0.44 ± 0.07	78.32 ± 5.59
2	7	25	5.0	157.0 ± 15.30	0.43 ± 0.04	73.28 ± 7.56
3	7	30	2.5	143.5 ± 16.43	0.08 ± 0.02	77.40 ± 4.88
4	9	20	5.0	120.9 ± 10.00	0.17 ± 0.05	87.84 ± 2.91
5	7	20	7.5	147.8 ± 15.77	0.10 ± 0.04	71.84 ± 2.74
6	7	20	2.5	161.6 ± 27.03	0.28 ± 0.05	7.000 ± 3.61
7	7	25	5.0	154.5 ± 15.93	0.50 ± 0.08	77.52 ± 5.61
8	9	30	5.0	129.3 ± 14.03	0.06 ± 0.04	87.52 ± 2.72
9	7	25	5.0	153.2 ± 20.05	0.43 ± 0.07	75.92 ± 5.59
10	9	25	7.5	139.8 ± 21.02	0.05 ± 0.02	87.52 ± 3.97
11	5	25	7.5	175.5 ± 19.33	0.59 ± 0.03	66.16 ± 5.91
12	5	25	2.5	199.5 ± 27.68	0.31 ± 0.06	60.16 ± 4.49
13	7	25	5.0	163.4 ± 2012	0.33 ± 0.05	76.64 ± 3.78
14	5	30	5.0	156.1 ± 15.53	0.05 ± 0.01	68.40 ± 5.59
15	7	25	5.0	156.9 ± 19.40	0.40 ± 0.04	74.72 ± 1.67
16	9	25	2.5	124.4 ± 12.64	0.15 ± 0.04	91.08 ± 2.95
17	5	20	5.0	189.7 ± 19.39	0.36 ± 0.04	63.84 ± 4.61

* Smix—surfactant and cosurfactant mixture; PDI—polydispersibility index; % EE—percentage entrapment efficiency. The data represented are mean ± SD (*n* = 3).

**Table 2:** Optimization of Que-NE by Box–Behnken design.

Source	Response 1 Droplet Size (nm)		Response 2 PDI *		Response 3 % EE	
ANOVA ANALYSIS						
	*F*-Value	*p*-Value	*F*-Value	*p*-Value	*F*-Value	*p*-Value
Model	43.55	<0.0001	3.52	0.0555	67.53	<0.0001
A-S_mix_	307.91	<0.0001	7.58	0.0284	194.99	<0.0001
B-% Amplitude	9.42	<0.0181	0.8011	0.4005	7.03	0.0199
C-Sonication time	0.8587	<0.3849	1.23	0.3036	0.5793	0.4602
AB	25.50	0.0015	0.7472	0.4160	-	-
AC	22.44	0.0021	2.91	0.1316	-	-
BC	9.25	0.0188	5.65	0.0491	-	-
A^2^	0.0019	0.9668	3.69	0.0963	-	-
B^2^	15.24	0.0059	7.58	0.0284	-	-
C^2^	2.03	0.1973	0.4818	0.5100	-	-
Lack of fit	1.28	0.3937	6.01	0.0580	2.61	0.1842
**Fit Statistics**						
R^2^	0.9825		0.8191		0.9397	
Adjusted R^2^	0.9599		0.5865		0.9258	
Adequate Precision	24.9500		5.8970		24.2220	
**Regression Coefficient Values**						
Intercept	157.00		0.4182		75.77	
A-S_mix_	−25.80		−0.1104		11.92	
B-% Amplitude	−4.51		−0.0359		2.27	
C-Sonication time	−1.36		0.0445		0.6500	
AB	10.50		0.0490		-	
AC	9.85		−0.0968		-	
BC	6.32		0.1348		-	
A^2^	−0.0875		−0.1061		-	
B^2^	−7.91		−0.1521		-	
C^2^	2.89		0.0384		-	

* PDI—polydispersibility index; % EE—percentage entrapment efficiency.

**Table 3:** Regression equations in coded terms for all the responses of Que-NE.

Response 1: Droplet size = +157.00 − 25.80 · A − 4.51 · B − 1.36 · C + 10.50 · A · B + 9.85 · A · C + 6.32 · B · C − 0.0875 · A^2^ − 7.91 · B^2^ + 2.89 · C^2^
Response 2: PDI = +0.4182 − 0.1104 · A − 0.0359 · B + 0.0445 · C + 0.0490 · A · B − 0.0968 · A · C + 0.1348 · B · C − 0.1061 · A^2^ − 0.1521 · B^2^ − 0.0384 · C^2^
Response 3: %EE = +75.77 + 11.92 · A + 2.27 · B + 0.6500 · C

**Table 4:** Predicted and experimental values for an optimized condition for Que-NE.

BBD-Optimized Condition		
Factors	Independent Variables	Actual Levels
A	S_mix_ ratio	9
B	% Amplitude	25
C	Sonication time (mins)	2.5
Responses	Predicted values	Experimental values
Droplet size (nm)	125.51	127.51 ± 7.71
PDI *	0.210	0.178 ± 0.02
EE (%) *	87.04	85.26 ± 4.69

* PDI—polydispersibility index; %EE—percentage entrapment efficiency.

**Table 5:** Pharmacokinetic parameters of Que-NE and Que-PD after oral administration (50 mg/kg BW).

Pharmacokinetic Parameter	Que-PD	Que-NE
T _max_ (h)	2 ± 0.0	4 ± 0.0
C_max_ (ng/mL)	1634.28 ± 70.18	5962.74 ± 238.54 **
C_48_ (ng/mL)	160.01 ± 38.93	798.62 ± 71.52 **
T_1/2_ (h)	21.79 ± 6.78	37.96 ± 7.74 *
AUC_0–48_ (ng/h/mL)	18,748.47 ± 930.16	83,667.94 ± 1610.30 **
AUC_0–∞_ (ng/h/mL)	24,079.09 ± 3556.69	128,205.28 ± 14677.35 **
Ke (h^−1^)	0.03 ± 0.00	0.01 ± 0.00 *
Vd (mL)	16,096 ± 2988.32	5279.78 ± 606.20 *
Cl (mL/h)	535.75 ± 97.77	98.02 ± 8.43 *
MRT (h)	28.78 ± 8.44	46.13 ± 9.91 *

Data presented are the mean ± standard deviation (*n* = 6). Significance was measured using one-way ANOVA. * *p* < 0.05, ** *p* < 0.01 effect of Que-NE on body weight (BW).

## Data Availability

Data is contained within the article.

## Supplementary Materials

The following supporting information can be downloaded at: https://www.mdpi.com/article/10.3390/ph15010070/s1, Figure S1: UV spectrum of Quercetin in methanol; Figure S2: Desirability Plot from Box-Behnken Design for Que-NE.

## References

[1] Davies M.J., Forni L.G., Willson R.L. (1988). Vitamin E analogue Trolox C. E.s.r. and pulse-radiolysis studies of free-radical reactions. Biochem. J..

[2] Shi G.J., Li Y., Cao Q.H., Wu H.X., Tang X.Y., Gao X.H., Yu J.Q., Chen Z., Yang Y. (2019). In vitro and in vivo evidence that quercetin protects against diabetes and its complications: A systematic review of the literature. Biomed. Pharmacother..

[3] Riva A., Ronchi M., Petrangolini G., Bosisio S., Allegrini P. (2019). Improved Oral Absorption of Quercetin from Quercetin Phytosome^®^, a New Delivery System Based on Food Grade Lecithin. Eur. J. Drug Metab. Pharmacokinet..

[4] Feeney O.M., Crum M.F., McEvoy C.L., Trevaskis N.L., Williams H.D., Pouton C.W., Charman W.N., Bergström C.A.S., Porter C.J.H. (2016). 50 years of oral lipid-based formulations: Provenance, progress and future perspectives. Adv. Drug Deliv. Rev..

[5] Kumar M., Bishnoi R.S., Shukla A.K., Jain C.P. (2019). Techniques for formulation of nanoemulsion drug delivery system: A review. Prev. Nutr. Food Sci..

[6] Kabri T., Arab-tehrany E., Belhaj N., Linder M. (2011). Physico-chemical characterization of nano- emulsions in cosmetic matrix enriched on omega-3. J. Nanobiotechnol..

[7] Komaiko J.S., Mcclements D.J. (2016). Formation of Food-Grade Nanoemulsions Using Low-Energy Preparation Methods: A Review of Available Methods. Compr. Rev. Food Sci. Food Saf..

[8] Aswathanarayan J.B., Vittal R.R. (2019). Nanoemulsions and Their Potential Applications in Food Industry. Front. Sustain. Food Syst..

[9] Matough F.A., Budin S.B., Hamid Z.A., Alwahaibi N., Mohamed J. (2012). The role of oxidative stress and antioxidants in diabetic complications. Sultan Qaboos Univ. Med. J..

[10] Ahmed S., Gull A., Alam M., Aqil M., Sultana Y. (2018). Ultrasonically tailored, chemically engineered and “QbD” enabled fabrication of agomelatine nanoemulsion; optimization, characterization, ex-vivo permeation and stability study. Ultrason. Sonochem..

[11] Qian C., McClements D.J. (2011). Formation of nanoemulsions stabilized by model food-grade emulsifiers using high-pressure homogenization: Factors affecting particle size. Food Hydrocoll..

[12] Son H.Y., Lee M.S., Chang E., Kim S.Y., Kang B., Ko H., Kim I.H., Zhong Q., Jo Y.H., Kim C.T. (2019). Formulation and characterization of quercetin-loaded oil in water nanoemulsion and evaluation of hypocholesterolemic activity in rats. Nutrients.

[13] Gokhale J.P., Mahajan H.S., Surana S.S. (2019). Quercetin loaded nanoemulsion-based gel for rheumatoid arthritis: In vivo and in vitro studies. Biomed. Pharmacother..

[14] Lotfi M., Kazemi S., Ebrahimpour A., Shirafkan F., Pirzadeh M., Hosseini M., Moghadamnia A.A. (2021). Protective Effect of Quercetin Nanoemulsion on 5-Fluorouracil-Induced Oral Mucositis in Mice. J. Oncol..

[15] Honary S., Ebrahimi P., Nikbakht M. (2013). Optimization of finasteride nano-emulsion preparation using chemometric approach. Trop. J. Pharm. Res..

[16] Ni S., Hu C., Sun R., Zhao G., Xia Q. (2017). Nanoemulsions-Based Delivery Systems for Encapsulation of Quercetin: Preparation, Characterization, and Cytotoxicity Studies. J. Food Process Eng..

[17] Myers R.H., Montgomery D.C., Anderson-Cook C.M. (2009). Response Surface Methodology Process and Product Optimization Using Designed Experiments.

[18] Pongsumpun P., Iwamoto S., Siripatrawan U. (2020). Response surface methodology for optimization of cinnamon essential oil nanoemulsion with improved stability and antifungal activity. Ultrason. Sonochem..

[19] Li Y., Fabiano-tixier A.S., Tomao V., Cravotto G., Chemat F. (2013). Ultrasonics Sonochemistry Green ultrasound-assisted extraction of carotenoids based on the bio-refinery concept using sunflower oil as an alternative solvent. Ultrason. Sonochem..

[20] Tran T.H., Guo Y., Song D., Bruno R.S., Lu X. (2014). Quercetin-containing self-nanoemulsifying drug delivery system for improving oral bioavailability. J. Pharm. Sci..

[21] Azeem A., Rizwan M., Ahmad F.J., Iqbal Z., Khar R.K., Aqil M., Talegaonkar S. (2009). Nanoemulsion components screening and selection: A technical note. AAPS PharmSciTech.

[22] Syed H.K., Peh K.K. (2014). Identification of phases of various oil, surfactant/co-surfactants and water system by ternary phase diagram. Acta Pol. Pharm.—Drug Res..

[23] Chen H., Chang X., Weng T., Zhao X., Gao Z. (2004). A study of microemulsion systems for transdermal delivery of triptolide. J. Control. Release.

[24] Abbas S., Bashari M., Akhtar W., Li W.W., Zhang X. (2014). Process optimization of ultrasound-assisted curcumin nanoemulsions stabilized by OSA-modified starch. Ultrason. Sonochem..

[25] Kumar S., Lather V., Pandita D. (2016). Stability indicating simplified HPLC method for simultaneous analysis of resveratrol and quercetin in nanoparticles and human plasma. Food Chem..

[26] Ke Z., Hou X., Jia X. (2016). bin Design and optimization of self-nanoemulsifying drug delivery systems for improved bioavailability of cyclovirobuxine D. Drug Des. Devel. Ther..

[27] Yang D.K., Kang H.S. (2018). Anti-diabetic effect of cotreatment with quercetin and resveratrol in streptozotocin-induced diabetic rats. Biomol. Ther..

[28] Friedewald W.T., Levy R.I., Fredrickson D.S. (1972). Estimation of the Concentration of Low-Density Lipoprotein Cholesterol in Plasma, Without Use of the Preparative Ultracentrifuge. Clin. Chem..

[29] Wu J., Yan L.J. (2015). Streptozotocin-induced type 1 diabetes in rodents as a model for studying mitochondrial mechanisms of diabetic β cell glucotoxicity. Diabetes Metab. Syndr. Obes. Targets Ther..

[30] Noeman S.A., Hamooda H.E., Baalash A.A. (2011). Biochemical study of oxidative stress markers in the liver, kidney and heart of high fat diet induced obesity in rats. Diabetol. Metab. Syndr..

[31] Kawakami K., Yoshikawa T., Hayashi T., Nishihara Y., Masuda K. (2002). Microemulsion formulation for enhanced absorption of poorly soluble drugs: II. In vivo study. J. Control. Release.

[32] Eaimtrakarn S., Rama Prasad Y.V., Ohno T., Konishi T., Yoshikawa Y., Shibata N., Takada K. (2002). Absorption enhancing effect of Labrasol on the intenstinal absorption of insulin in rats. J. Drug Target..

[33] Wang-Fischer Y., Garyantes T. (2018). Improving the reliability and utility of streptozotocin-induced rat diabetic model. J. Diabetes Res..

[34] Elsheikh M.A., Elnaggar Y.S.R., Gohar E.Y., Abdallah O.Y. (2012). Nanoemulsion liquid preconcentrates for raloxifene hydrochloride: Optimization and in vivo appraisal. Int. J. Nanomed..

[35] Thakore S., Patel R., Patel M. (2014). Nanoemulsion or Microemulsion?—Understanding the Differences and Similarities. Pharma Rev..

[36] Mazonde P., Khamanga S.M.M., Walker R.B. (2020). Design, optimization, manufacture and characterization of Efavirenz-loaded flaxseed oil nanoemulsions. Pharmaceutics.

[37] Kale S.N., Deore S.L. (2016). Emulsion micro emulsion and nano emulsion: A review. Syst. Rev. Pharm..

[38] Carpenter J., Saharan V.K. (2017). Ultrasonic assisted formation and stability of mustard oil in water nanoemulsion: Effect of process parameters and their optimization. Ultrason. Sonochem..

[39] Taylor P., Homayoonfal M., Khodaiyan F., Mousavi S.M., Homayoonfal M., Khodaiyan F., Mousavi S.M. (2014). Walnut Oil Nanoemulsion: Optimization of the Emulsion Capacity, Cloudiness, Density, and Surface Tension. J. Dispers. Sci. Technol..

[40] D’Souza S. (2014). A Review of In Vitro Drug Release Test Methods for Nano-Sized Dosage Forms. Adv. Pharm..

[41] Hou J., Han M., Wang J. (2021). Manipulation of surface charges of oil droplets and carbonate rocks to improve oil recovery. Sci. Rep..

[42] Shah B.M., Misra M., Shishoo C.J., Padh H. (2015). Nose to brain microemulsion-based drug delivery system of rivastigmine: Formulation and ex-vivo characterization. Drug Deliv..

[43] Lv L., Liu C., Li Z., Song F., Li G., Huang X. (2017). Pharmacokinetics of Quercetin-Loaded Methoxy Poly(ethylene glycol)-b-poly(L-lactic acid) Micelle after Oral Administration in Rats. Biomed Res. Int..

[44] Li H.L., Zhao X.B., Ma Y.K., Zhai G.X., Li L.B., Lou H.X. (2009). Enhancement of gastrointestinal absorption of quercetin by solid lipid nanoparticles. J. Control. Release.

[45] Hussain N., Jaitley V., Florence A.T. (2001). Recent advances in the understanding of uptake of microparticulates across the gastrointestinal lymphatics. Adv. Drug Deliv. Rev..

[46] Venkatesan N., Uchino K., Amagase K., Ito Y., Shibata N., Takada K. (2006). Gastro-intestinal patch system for the delivery of erythropoietin. J. Control. Release.

[47] Lim S.J., Lee M.K., Kim C.K. (2004). Altered chemical and biological activities of all-trans retinoic acid incorporated in solid lipid nanoparticle powders. J. Control. Release.

[48] Salehi B., Machin L., Monzote L., Sharifi-Rad J., Ezzat S.M., Salem M.A., Merghany R.M., El Mahdy N.M., Klllç C.S., Sytar O. (2020). Therapeutic Potential of Quercetin: New Insights and Perspectives for Human Health. ACS Omega.

[49] Yim S., Malhotra A., Veves A. (2007). Antioxidants and CVD in diabetes: Where do we stand now?. Curr. Diab. Rep..

[50] Ragavan G., Muralidaran Y., Sridharan B., Nachiappa Ganesh R., Viswanathan P. (2017). Evaluation of garlic oil in nano-emulsified form: Optimization and its efficacy in high-fat diet induced dyslipidemia in Wistar rats. Food Chem. Toxicol..

[51] Sangeetha M.K., Eazhisai Vallabi D., Sali V.K., Thanka J., Vasanthi H.R. (2013). Sub-acute toxicity profile of a modified resveratrol supplement. Food Chem. Toxicol..

[52] Chen P., Chen J., Zheng Q., Chen W., Wang Y., Xu X. (2013). Pioglitazone, extract of compound Danshen dripping pill, and quercetin ameliorate diabetic nephropathy in diabetic rats. J. Endocrinol. Investig..

