# Supplementary material for: Fabrication and Evaluation of Quercetin Nanoemulsion: A Delivery System with Improved Bioavailability and Therapeutic Efficacy in Diabetes Mellitus
Source: Pharmaceuticals (Basel). 2022 Jan 5;15(1):70. doi: 10.3390/ph15010070 (PMC8779357; doi:10.3390/ph15010070)
Supplement: Supplementary file 1 [file pharmaceuticals-15-00070-s001.zip › Supplementary Figure S2.pdf]

Design-Expert® Software
Factor Coding: Actual

All Responses
Design Points
0.000 1.000

Desirability = 1.000
Std # 6 Run # 14

X1 = A: Smix = 9
X2 = B: Amplitude = 25

Actual Factor
C: Sonication Time = 2.5

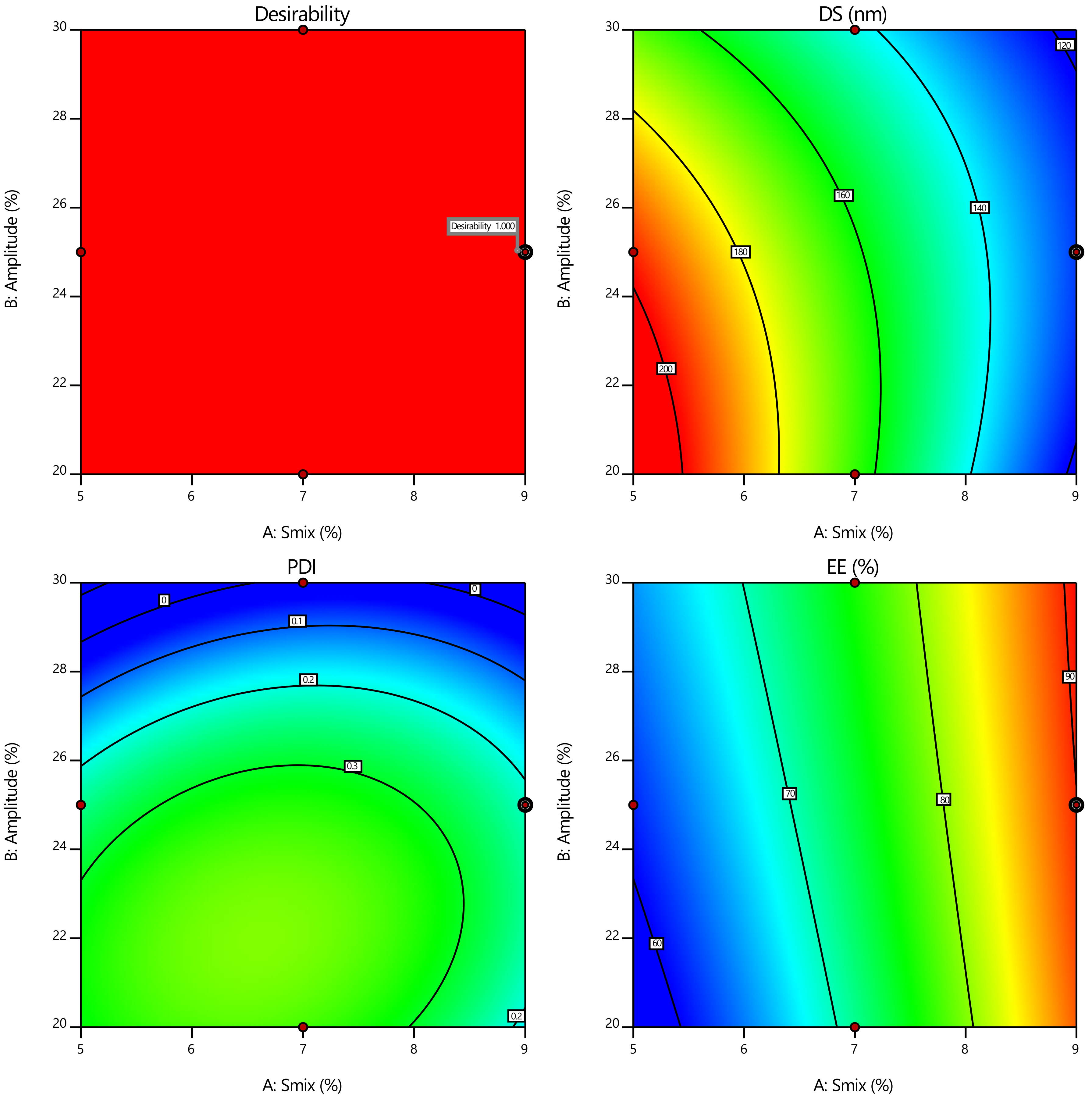

Supplementary Figure S2: Desirability Plot from Box-Behnken Design for Que-NE
