# Supplementary figures and images for: Fabrication and Evaluation of Quercetin Nanoemulsion: A Delivery System with Improved Bioavailability and Therapeutic Efficacy in Diabetes Mellitus
Source: Pharmaceuticals (Basel). 2022 Jan 5;15(1):70. doi: 10.3390/ph15010070 (PMC8779357; doi:10.3390/ph15010070)

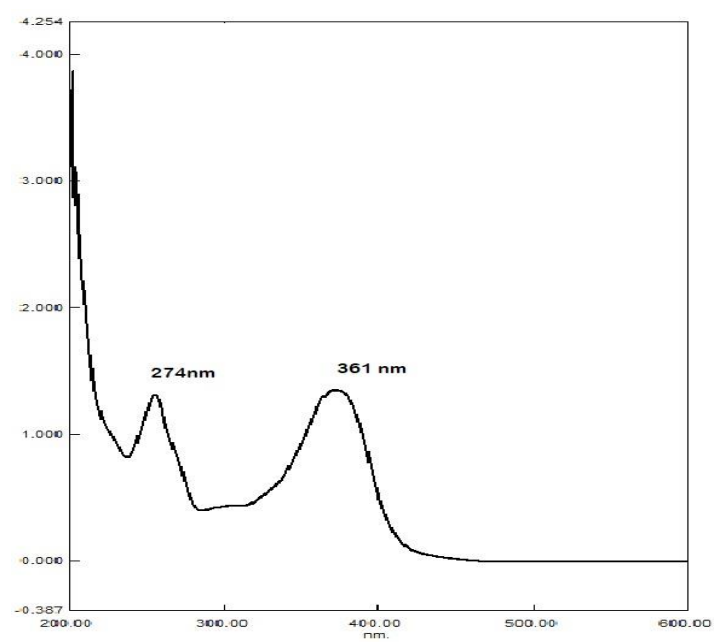

Supplementary Figure S1. UV spectrum of Quercetin in methanol

Supplement: Supplementary file 1 [file pharmaceuticals-15-00070-s001.zip › Supplementary Figure S1.pdf]
